# Fecal Capsule as a Therapeutic Strategy in IgA Nephropathy: A Brief Report

**DOI:** 10.3389/fmed.2022.914250

**Published:** 2022-05-12

**Authors:** Wenqiang Zhi, Wenzhu Song, Yasin Abdi Saed, Yi Wang, Yafeng Li

**Affiliations:** ^1^Department of Nephrology, The Fifth Hospital (Shanxi Provincial People’s Hospital) of Shanxi Medical University, Taiyuan, China; ^2^School of Public Health, Shanxi Medical University, Taiyuan, China; ^3^The Third Clincial College, Shanxi University of Chinese Medicine, Taiyuan, China; ^4^Shanxi Provincial People’s Hospital, Shanxi Provincial Key Laboratory of Kidney Disease, Taiyuan, China; ^5^Academy of Microbial Ecology, Shanxi Medical University, Taiyuan, China

**Keywords:** IgA nephropathy, fecal microbiota transplantation, gut microbiota, FMT capsule, 24-h urine protein quantification

## Abstract

In this brief report, we reported an IgA nephropathy (IgAN) patient who presented in November 2020 with an acute exacerbation with massive proteinuria and diarrhea. He had the earliest onset in 2018 when his IgAN was diagnosed by renal biopsy. He has been treated with active ACEI/ARB drugs for more than 90 days, intermittent steroid therapy, combined with anti-infective therapy. Although his acute symptoms resolved with each episode, he became increasingly severe as the interval between episodes shortened. Accordingly, the immunosuppressive drugs were administered under the KDIGO guidelines and related guidelines. However, the patient and his family refused this treatment. We pondered over the possible pathogenesis of IgAN, and after a full discussion with the patient and his family, FMT was administered to him after obtaining his informed consent. During the FMT procedure, one healthy volunteer (the doctor himself) also took the FMT capsules. In the end, the patient’s urine protein dropped significantly and even turned negative after treatment. Neither the patient nor the healthy volunteer experienced any serious adverse effects during the use of the capsules and the subsequent 6-month follow-up period. We also used metagenomic sequencing to analyze the intestinal flora of patients before and after treatment, and a gradual increase stood out in the abundance of the patient’s intestinal flora after drug administration.

## Introduction

IgA nephropathy (IgAN) is the most common primary glomerular disease worldwide and the leading cause of chronic renal insufficiency and renal failure (1). Approximately 25% of patients with IgAN would be subject to end-stage renal disease (ESRD), which requires renal replacement therapy. It has emerged as a major public health issue globally (2). Its pathogenesis remains to be elucidated, and there’s no specific and effective treatments are available, except for optimized supportive care.

## Diagnosis and Treatment Process

### Case Presentation

Herein, we report a case of a 21-year-old man who repeatedly visited the Department of Nephrology of Shanxi Provincial People’s Hospital for hematuria with diarrhea.

The patient initially presented to the emergency department in 2018 with no obvious cause of tea-colored, foamy urine, occasional burning sensation, intermittent lumbar pain. He was examined for a 24-h urine protein, which stood at 3.19 g/24 h, and his blood pressure is 128/76 mmHg. An ultrasound-guided renal biopsy was performed on the second day of admission to clarify the cause of hematuria and proteinuria and the type of renal pathology.

Pathological reports revealed: Fourteen glomeruli, mesangial cells, and mesangial matrix were mildly segmentally hyperplastic with metanephrin deposition, and 1 small cellular crescent. Vacuolar degeneration of renal tubular epithelium, renal interstitium and arterioles showed no definite lesions. Immunofluorescence sections showed no glomeruli, and immunofluorescence with supplemental paraffin: IgG (–) IgA (+) IgM (–) C3 (–) FRA (–) C1q (–) was deposited in mesangial areas in a granular pattern. Immunohistochemical results: HBsAg (–), HBcAg (–), kappa (+), lambda (+). Consider mild mesangial proliferative IgA nephropathy, M0E0S0T0C1. Information on pathology pictures is provided in Supplementary Material.

Urine routine showed urine protein 1+, erythrocytes 2+, microscopy erythrocytes 45–50/HP, erythrocyte variability 85%. Prednisone 20 mg/day and Irbesartan 150 mg/day were given to the patient. Prednisone was reduced by 10 mg every 2 months, and further reduced to 5 mg daily for maintenance. 6 months later, the urine protein quantification was rechecked at 0.3 g/24 h with hematuria ±, so the patient stopped hormone intake.

Two years later, the patient had a fever with no apparent cause, with a maximum temperature of 37.6°C. He was readmitted to the Department of Nephrology for hematuria. The examination showed that urine red blood cells were 3+, urine protein was 3+, 24-h urine protein quantification was 3.83 g/24 h. As such, the patient received 5 g of piperacillin sulbactam and 0.1 g/day of anti-infective hydroxychloroquine. With improved symptoms, he was then discharged with regular oral prednisone acetate tablets, which were discontinued after 2 months.

In November 2020, the patient was again admitted to the hospital with meatus hematuria and with dilute stools 4–5 times/day. Clinical examination showed urine protein quantification 2.83 g/24 h, and urine routine examination showed red blood cells 1+, urine protein 3+. Therefore, 5 g of piperacillin sulbactam and 0.1 g/day of anti-infective hydroxychloroquine were given to the patient. Irbesartan 150 mg/day was used for protein reduction. Four days later, the 24-h urine protein quantification reached 4.98 g/24 h.

### Fecal Microbiota Transplantation Procedure

Donor screening and FMT capsule preparation were conducted by Dongyuan Yikang, Beijing, China. Donors were first clinically evaluated by questionnaire, including medical history, behavioral risk, and current health status. Potential candidates are then screened fecally and serologically to rule out infectious diseases and potential intestinal dysregulation-related diseases. After screening, 2–3% were eligible enough to be donors. The fresh feces collected from donors were subsequently diluted, filtered, and centrifuged by professional technicians in Dongyuan Yikang’s clean laboratory to produce FMT capsules. The patient started the FMT after antibiotic consumption for 1 month. Afterward, FMT capsules were given on days 1, 8, and 15 for one course of treatment. The patient continued to take irbesartan during the FMT treatment.

## Results

**FIGURE 1:**
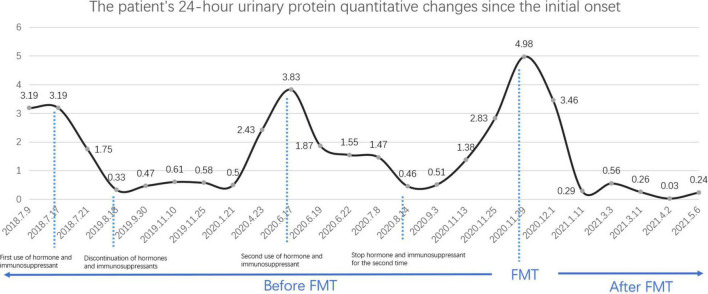
Patient’s 24-h urine protein quantitative changes since the initial onset.

This patient was followed up for 6 months after treatment and showed a significant downward trend in 24-h urine protein quantification, which is a validated prognostic biomarker. It first decreased to 0.29 g/24 h 1 month after FMT. Surprisingly, his 24-h urine protein quantification eventually witnessed a negative conversion 3 months after FMT treatment (Figure 1).

**FIGURE 2:**
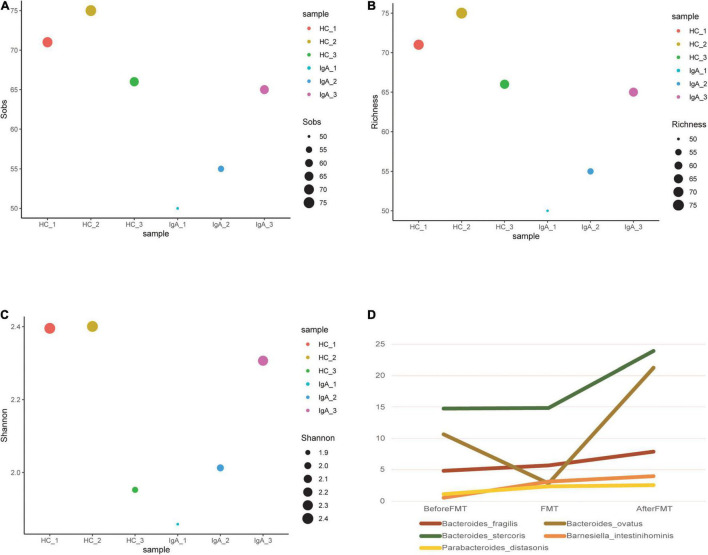
Changes in alpha diversity and main bacterial species before and after FMT. **(A–C)** Were to show the diversity of IgAN patient and healthy control in this study. **(A,B)** Were to reflect the Richness, and **(C)** was to show the Evenness. The larger the point, the larger the value is. Different color means different samples before or after FMT. **(D)** Was to reflect the changes of main bacterial strains.

We also used metagenomic sequencing to analyze the intestinal flora of patients before and after treatment. Alpha diversity is to describe the structure of an ecological community with regard to its richness (number of taxonomic groups), evenness (distribution of abundances of the groups), or both (3). Sobs index and Richness index are to show its richness, whereas Shannon index is to reflect its evenness. Figures 2A–C showed us the gradual increase of alpha-diversity, which was close to the healthy control, indicating the recovery of intestinal composition. The healthy control exhibited no obvious change but slight fluctuation. Meanwhile, no discomfort or side effects were observed, which, in a sense, demonstrated the efficacy and safety of the FMT capsule.

In general, a gradual increase stood out in the abundance of the patient’s intestinal flora after drug administration. However, some bacterial abundance were on the increase, such as Bacteroides_fragilis, Bacteroides_ovatus and Bacteroides_stercoris, and some were on the wane, including Eggerthella_lenta, Bilophila_wadsworthia, and Escherichia_coli (Figure 2D).

## Discussion

New discoveries suggest that the pathogenesis of IgAN involves excessive circulation of gd-IgA which is target of anti-glycan antibodies. Immune complexes formed by gd-IgA and anti-glycan antibodies ultimately deposit in the mesangium, causing mesangial cell proliferation, secretion of pro-inflammatory cytokines and complement activation (4). More questions are being asked about the origin of focal aberrant IgA, mainly in the mucosal immune response. Previous studies have focused on the hyperresponsiveness of the upper respiratory tract and tonsil mucosa, but current studies exhibit that altered gut microbial composition and immune and metabolic dysregulation of the gut may have a combined role in the development of IgAN through the intestinal-renal axis mechanism, a hypothesis that also provides a theoretical support for the treatment of IgAN by regulation of gut microbial composition. Some studies demonstrated that risk genotypes concerning gut microbial composition are associated with higher serum galactose-deficient IgA1 (GdIgA1) levels (5). Related studies have revealed that mucosal infections can cause acute episodes of IgAN (6), that changes in the microbiota are also associated with the severity of IgAN (7, 8).

Based on the current study, we believe that the future treatment of IgAN will be primarily targeted at the mucosal microbiota, together with some novel agents (such as hydroxychloroquine). Fecal microbiota transplantation (FMT), which involves functional flora transplantation into a diseased gut microbiota, helps to re-establish a normal intestinal flora composition and has been used in various intestinal diseases such as CDI with good efficacy and safety (9). After we performed FMT in this patient, his clinical symptoms were dramatically relieved, the intestinal microbial diversity was significantly increased, which undoubtedly provides a new idea for the treatment of IgAN.

Since the first episode in 2008, the patient has been treated with irbesartan at a starting dose of 150 mg/day and has maintained this dose for a long period, including during each acute episode and FMT implementation. On first admission, the patient’s blood pressure fluctuated at 113/68 mmHg–135/78 mmHg with a maximum of 135/78 mmhg only once. Considering the patient’s good pressure control, the physician did not treat this patient at the maximum dose. The patient discontinued hormonal therapy and anti-infective therapy in the middle of the disease course. Initially, significant results were obtained with hormonal and anti-infective therapy, but in the subsequent course, patients had shorter intervals between each acute episode, more severe infectious symptoms and increased proteinuria. After the first hormonal and anti-infective treatment, the patient was in remission and maintained so for 23 months. However, the most recent acute episode was only 3 months after previous episode, and the infection symptoms were significantly aggravated, accompanied by sore throat, expectoration, diarrhea 4–5 times/day in the form of loose stools. Before admission, he was self-administered amoxicillin 1 g/dose, 3 times/day, orally. There was an improvement in diarrhea symptoms but no significant improvement in hematuria. Antibiotic therapy was empirically used by the physician after admission, and irbesartan was maintained. Five days after admission, the patient’s symptoms of hematuria and sore throat and diarrhea improved, but proteinuria was not effectively controlled and appeared progressively elevated. Seventeen days after discontinuation of antibiotics, the patient was treated with FMT.

In terms of gut microbiota, the patient presented with an increased intestinal flora diversity after FMT, accompanied by an increase of Clostridium_symbiosum, a bacterium whose abundance is positively correlated with renal ACE2 expression (10). Yet, ACE2 deficiency is associated with renal impairment, renal fibrosis, and other kidney-related diseases. Of note, Bacteroides_ovatus has also increased after FMT; It was found that different bacterial strains also vary in the induction of IgA (11). It remains unclear whether the increased Bacteroides_ovatus would induce more IgA production in humans. More follow-up studies are needed, and our ongoing recruitment of IgAN patients would shed light on the alteration of this bacterial strain.

FMT has now been applied to a variety of diseases associated with intestinal dysbioses, such as CDI, IBD, IBS, obesity, and diabetes; it shows efficacy and safety in the above different diseases (12), but a paucity of research on its use in IgAN has been carried out. The patient in our study was treated with FMT capsules, a simpler procedure with lower incidence of side effects, lower risk of bleeding or perforation by endoscopic operation and thus higher acceptance, compared with traditional enema or endoscopy. After FMT, the patient’s proteinuria turned negative, with no significant side effects during treatment and follow-up, showing good efficacy and safety.

In this report, limitation also emerges that more clinical evidence is required to prove the efficacy and safety of FMT in IgAN. Regarding other indicators reflecting prognosis, we didn’t conduct a regular monthly test for them, such as serum creatinine, as there were no significant abnormalities in the patient’s renal function or serum albumin. As such, the effect of FMT on these indicators still needs to be further verified. Meanwhile, with regard to the timing of FMT capsule use as well as usage, we previously referenced the standard usage provided by the capsule preparer, and the optimal usage for patients with IgA still needs further validation. Since, we are preparing for a single-center clinical trial, whose protocol was approved by the ethics committee of Shanxi Provincial People’s Hospital. Besides, we have managed to register the protocol in Chinese Clinical Trial Registry Center (ChiCTR2100053206), and in ClinicalTrials.gov (NCT05182775).

## Data Availability Statement

The original contributions presented in the study are included in the article/Supplementary Material, further inquiries can be directed to the corresponding author/s.

## Ethics Statement

Written informed consent was gained from the patient and the treatment was approved by the ethics committee for clinical trials of Shanxi Provincial People’s Hospital.

## Author Contributions

WZ drafted the original manuscript. WS helped polish the manuscript and responsible for the Figure. YA and YW revised the manuscript. YL gave the overall design of this manuscript. All authors contributed to the article and approved the submitted version.

## Conflict of Interest

The authors declare that the research was conducted in the absence of any commercial or financial relationships that could be construed as a potential conflict of interest.

## Publisher’s Note

All claims expressed in this article are solely those of the authors and do not necessarily represent those of their affiliated organizations, or those of the publisher, the editors and the reviewers. Any product that may be evaluated in this article, or claim that may be made by its manufacturer, is not guaranteed or endorsed by the publisher.

## References

[1] Suzuki H Novak J. IgA glycosylation and immune complex formation in IgAN. *Semin Immunopathol.* (2021) 43:669–78. 10.1007/s00281-021-00883-8 34570260

[2] Jarrick S Lundberg S Welander A Carrero JJ Höijer J Bottai M Mortality in IgA nephropathy: a nationwide population-based cohort study. *J Am Soc Nephrol.* (2019) 30:866–76. 10.1681/ASN.2018101017 30971457 PMC6493992

[3] Willis AD. Rarefaction, alpha diversity, and statistics. *Front Microbiol.* (2019) 10:2407. 10.3389/fmicb.2019.02407 31708888 PMC6819366

[4] Lai KN Tang SC Schena FP Novak J Tomino Y Fogo AB IgA nephropathy. *Nat Rev Dis Primers.* (2016) 2:16001. 27189177 10.1038/nrdp.2016.1

[5] He JW Zhou XJ Li YF Wang YN Liu LJ Shi SF Associations of genetic variants contributing to gut microbiota composition in immunoglobin a nephropathy. *mSystems.* (2021) 6:e819–20. 10.1128/mSystems.00819-20 33436510 PMC7901477

[6] Selvaskandan H Barratt J Cheung CK. Immunological drivers of IgA nephropathy: exploring the mucosa-kidney link. *Int J Immunogenet.* (2022) 49:8–21. 10.1111/iji.12561 34821031

[7] Watanabe H Goto S Mori H Higashi K Hosomichi K Aizawa N Comprehensive microbiome analysis of tonsillar crypts in IgA nephropathy. *Nephrol Dial Transplant.* (2017) 32:2072–9. 10.1093/ndt/gfw343 27683270

[8] Cao Y Qiao M Tian Z Yu Y Xu B Lao W Comparative analyses of subgingival microbiome in chronic periodontitis patients with and without IgA nephropathy by high throughput 16S rRNA sequencing. *Cell Physiol Biochem.* (2018) 47:774–83. 10.1159/000490029 29807361

[9] Wang JW Kuo CH Kuo FC Wang YK Hsu WH Yu FJ Fecal microbiota transplantation: review and update. *J Formos Med Assoc.* (2019) 118(Suppl 1):S23–31. 10.1016/j.jfma.2018.08.011 30181015

[10] Snelson M Muralitharan RR Dinakis E Nakai M Jama HA Shihata WA Renal ACE2 (Angiotensin-Converting Enzyme 2) expression is modulated by dietary fiber intake, gut microbiota, and their metabolites. *Hypertension.* (2021) 77:e53–5. 10.1161/HYPERTENSIONAHA.121.17039 33866801

[11] Yang C Mogno I Contijoch EJ Borgerding JN Aggarwala V Li Z Fecal IgA levels are determined by strain-level differences in *Bacteroides ovatus* and are modifiable by gut microbiota manipulation. *Cell Host Microbe.* (2020) 27:467–75.e466. 10.1016/j.chom.2020.01.016 32075742 PMC7213796

[12] Aroniadis OC Brandt LJ. Fecal microbiota transplantation: past, present and future. *Curr Opin Gastroenterol.* (2013) 29:79–84. 10.1097/mog.0b013e32835a4b3e 23041678

