# Supplementary figures and images for: Fecal Capsule as a Therapeutic Strategy in IgA Nephropathy: A Brief Report
Source: Front Med (Lausanne). 2022 May 12;9:914250. doi: 10.3389/fmed.2022.914250 (PMC9133370; doi:10.3389/fmed.2022.914250)

*Supplementary Material*

pathology figures1.

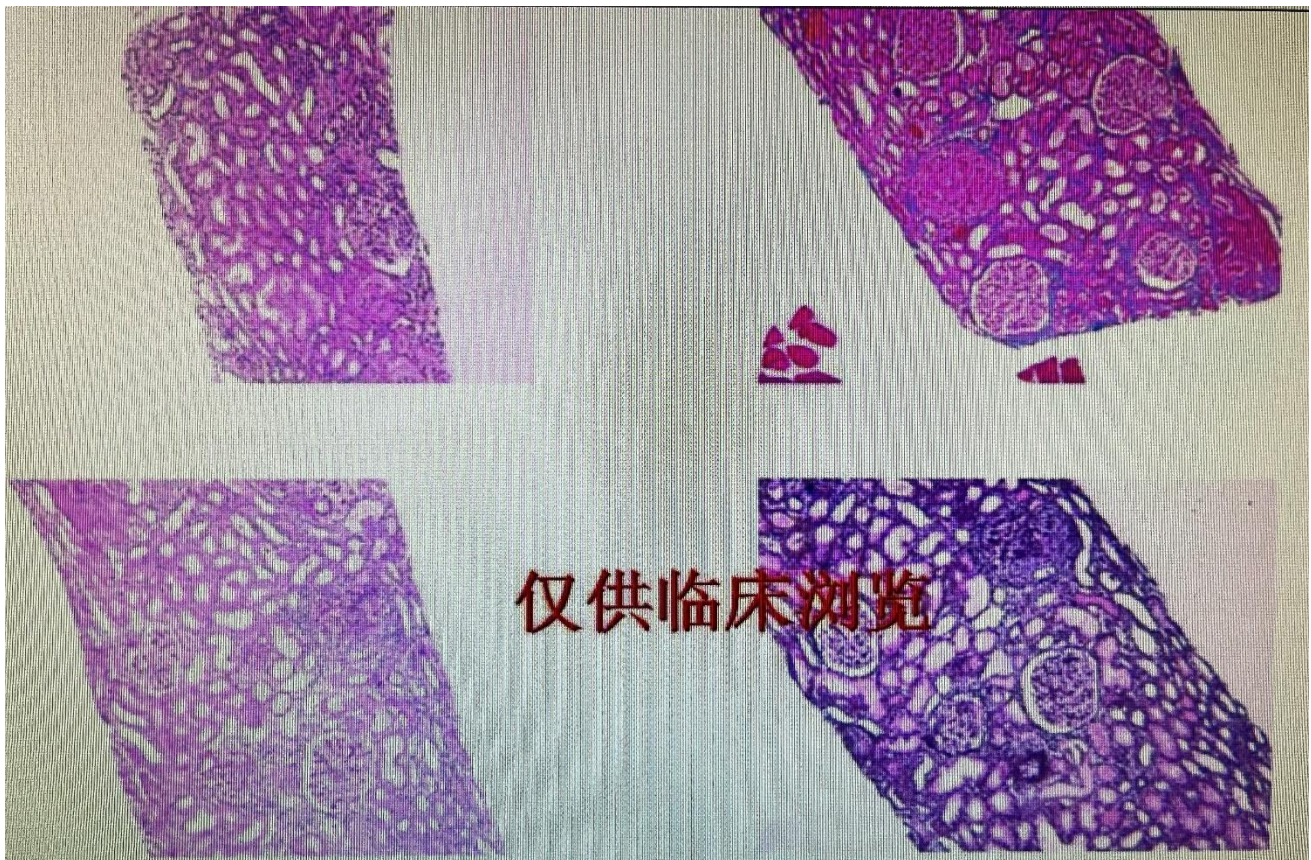

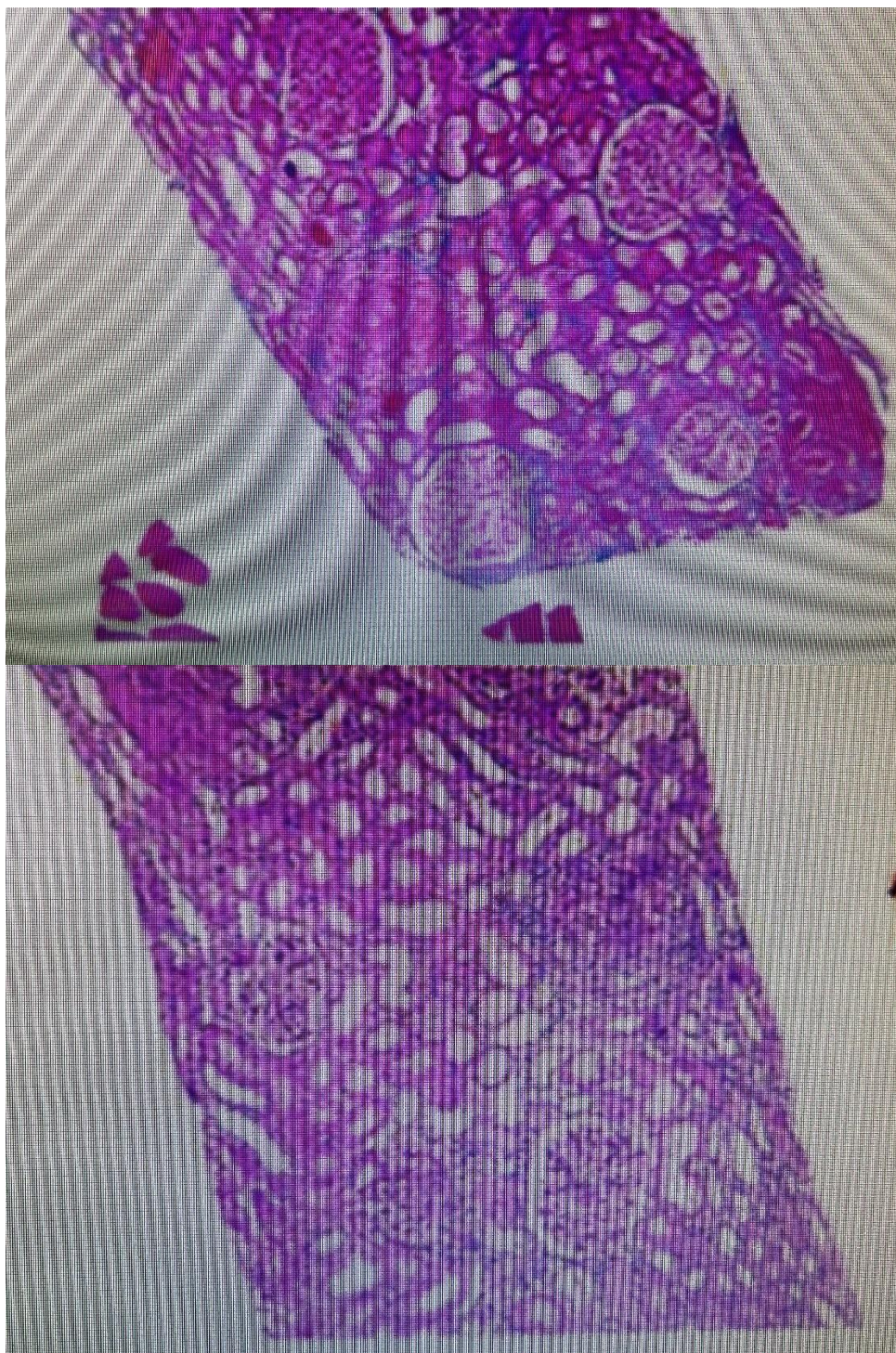

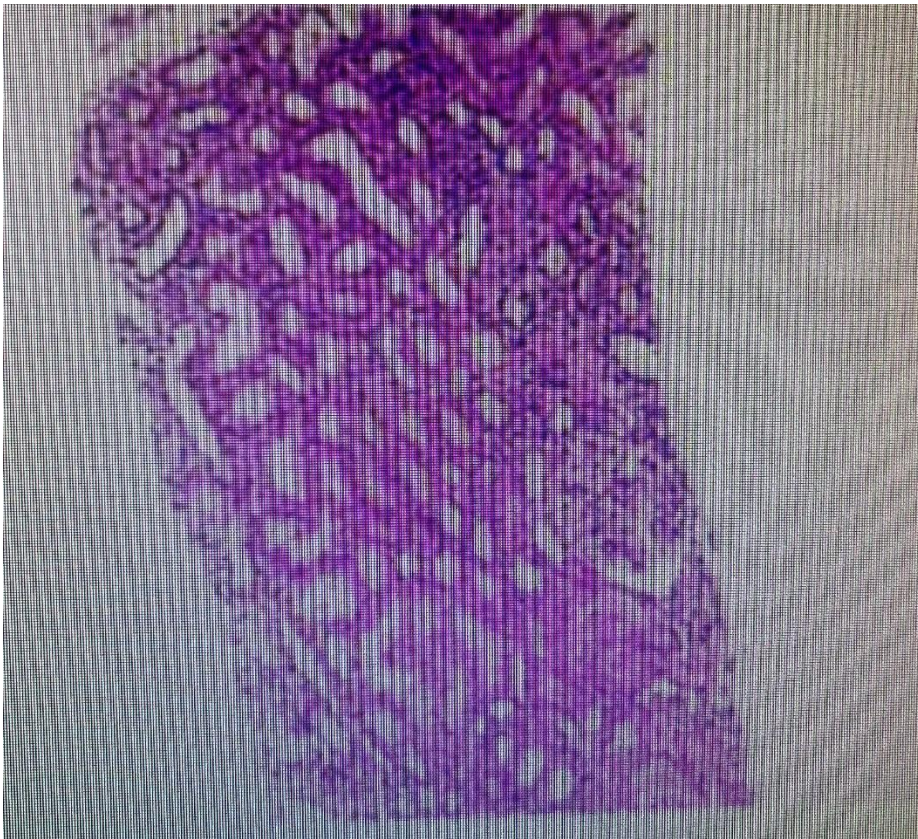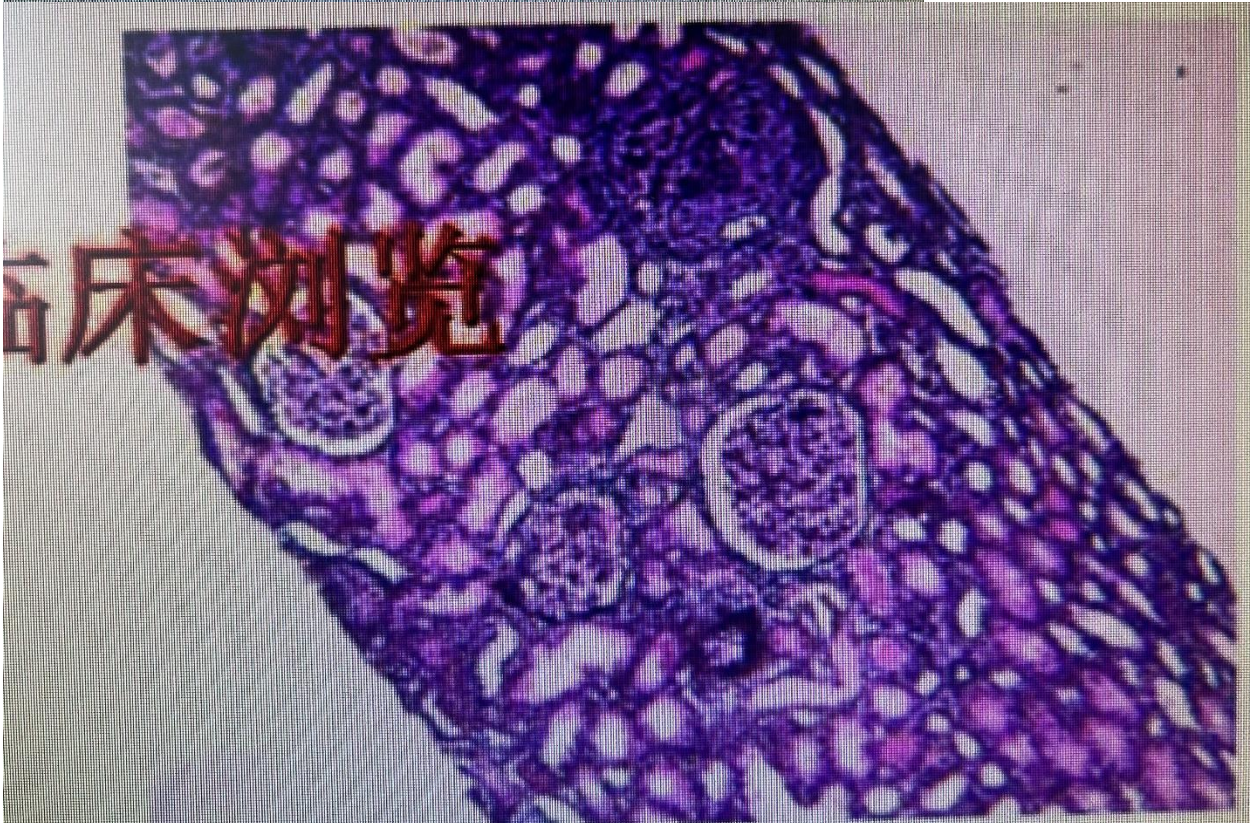

Supplement: Supplementary file 1 [file Data_Sheet_1.pdf]
